# Editorial: The role of gut microbiota in immune-related inflammatory diseases

**DOI:** 10.3389/fcimb.2026.1873260

**Published:** 2026-05-25

**Authors:** Cinzia Parolini, Núria Mach

**Affiliations:** 1 Department of Pharmacological and Biomolecular Sciences, “Rodolfo Paoletti”, Università degli Studi di Milano, Milano, Italy; 2 Univ Toulouse, École Nationale Vétérinaire de Toulouse (ENVT), Institut National de Recherche pour l’Agriculture, l’Alimentation et l’Environnement (INRAE), Interactions Hôtes–Agents Pathogènes (IHAP), Toulouse, France

**Keywords:** dysbiosis, gut microbiota, immune modulation, inflammatory disease, microbial metabolites

Over the past decade, the gut microbiota has emerged as one of the most powerful regulators of host homeostasis. No longer viewed as a passive collection of commensals, it is now recognized as a dynamic immunological and endocrine interface that shapes metabolism, immune function, and systemic inflammation from early life through disease. Dysbiosis—whether through altered microbial composition or disrupted metabolic output—has been implicated in a wide range of immune-mediated inflammatory diseases, including inflammatory bowel disease, diabetes, psoriasis, allergic disorders, rheumatoid arthritis, and systemic lupus erythematosus. Yet fundamental questions remain: Which microbial taxa act as true causal drivers? Which metabolites function as key immunomodulators? How can these insights be translated into reliable biomarkers or targeted microbiota-based therapies?

This Research Topic brings together five complementary studies that collectively illuminate how gut microbes and their metabolites influence inflammatory diseases across organs. The first contribution by Zheng et al. reveals a dynamic gut–immune–kidney axis in diabetic kidney disease (DKD). As DKD progresses, gut microbial composition shifts markedly and CD4^+^ T cells emerge as critical intermediaries linking dysbiosis to renal injury. Patients with reduced CD4^+^ T cell count display distinct microbial signatures and poorer clinical outcomes, suggesting that immune profiling may help identify individuals at risk of accelerated DKD progression. Extending the theme of microbe–metabolite–immune interactions, Wang et al. show that diet-induced dampness-heat psoriasis is driven by a characteristic dysbiosis—most notably the loss of *Lactobacillus*—and a profound disruption of bile acid metabolism. Elevated deoxycholic acid, altered FXR/CYP7A1 signaling, and hepatic lipid deposition reveal how diet, microbial metabolites, and cutaneous inflammation intersect along the gut–skin axis. Their findings highlight bile acid imbalance and *Lactobacillus* depletion as mechanistic contributors to psoriasis exacerbation. To situate these mechanistic insights within the broader scientific landscape, Zou et al. provide a bibliometric analysis charting two decades of research on psoriasis and the gut microbiota. Their work reveals a shift from early associative studies to a mechanistic focus on gut barrier dysfunction, short-chain fatty acid metabolism, Treg impairment, and Th17 activation. By mapping emerging hotspots and future priorities, they reinforce the central role of gut dysbiosis in systemic and skin inflammation and outline promising directions for microbiota-based diagnostics and therapies.

Using high-resolution microbial profiling, Orejudo et al. characterized the microbiota of patients with newly diagnosed, treatment-naïve inflammatory bowel disease (IBD). Their shotgun metagenomic analysis uncovers pronounced early dysbiosis in Crohn’s disease, including reduced species diversity and shifts in low-abundance but influential taxa such as *Gemella*, *Adlercreutzia*, and *Toxoplasma gondii*.

Finally, asthma, an inflammatory lung disease, affects women in adulthood. Menopause is often linked to the onset or worsening of preexisting asthma. To study asthma symptoms during menopause, Perez Umana et al. used a mouse model of postmenopausal asthma via ovariectomy (OVx). They demonstrated that preventive acetate treatment protects OVx mice from exacerbated allergic airway inflammation. Acetate reduces type 2 lung responses, increases regulatory T cells, and restores intestinal epithelial markers (*MUC2*, *OCLN*), revealing a mechanistic link among estrogen decline, gut barrier integrity, and airway inflammation. This study underscores the potential of microbial metabolites as cross-organ immunomodulators.

**Figure 1:**
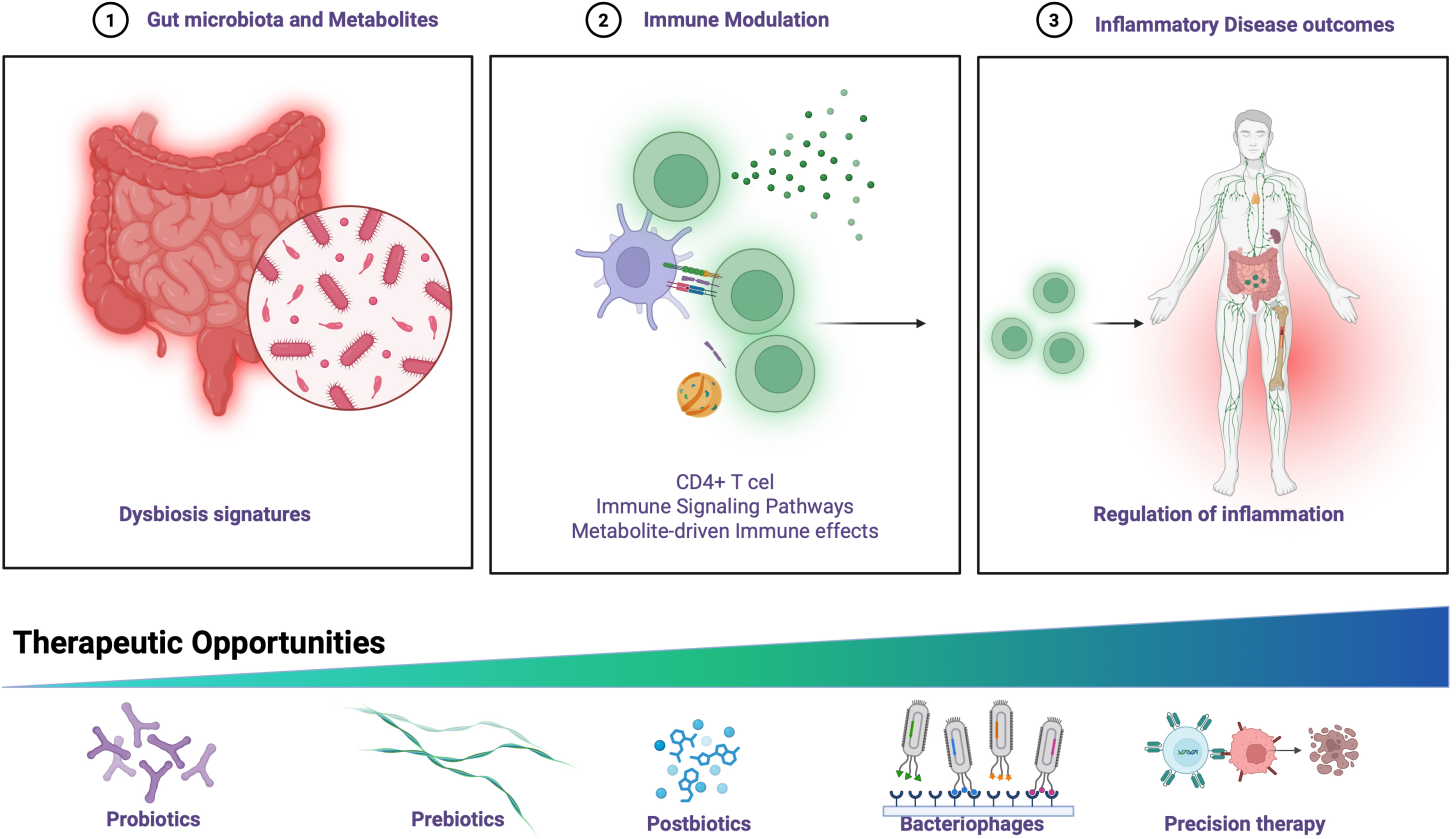
Gut microbiota-immune interplay in immune-related inflammatory diseases and therapeutic opportunities. Schematic representation of how the gut microbiota and its metabolites influence host physiology. Left: Dysbiosis signatures arising from alterations in microbial composition and metabolic outputs. Center: Immune modulation, including metabolite-driven effects on immune signaling pathways and CD4^+^ T cell responses. Right: Consequences for inflammatory disease outcomes, highlighting the regulation of intestinal and systemic inflammation. Bottom: Overview of emerging therapeutic strategies targeting the microbiota–immune axis, including probiotics, prebiotics, postbiotics, bacteriophages, and precision therapies.

Together, these studies illustrate the breadth and depth of the microbiota-gut–immune axis, showing how microbial communities and their metabolites influence inflammation across the kidney, skin, intestine, and lungs (**Figure 1**). They collectively underscore the promise of microbiota-targeted strategies for understanding, diagnosing, and treating immune-mediated inflammatory diseases.

Several new hypotheses emerge from this body of work. One is that specific immune cell subsets—such as CD4^+^ T cells, or tissue-resident lymphocytes—may act as intermediaries that translate microbial signals into systemic inflammatory responses. Another is that microbial metabolites, particularly short-chain fatty acids and bile acid derivatives, may function as endocrine-like molecules, with effects shaped by host metabolic and hormonal status or by diet. A third hypothesis is that early dysbiosis signatures in diseases such as IBD or psoriasis may not simply reflect inflammation but could represent preclinical microbial shifts that predispose individuals to disease onset.

Advancing the field will require integrating metagenomics, metabolomics, immunophenotyping, and host genetics and epigenetics into unified frameworks. It will also require expanding microbiota-based interventions beyond traditional probiotics and prebiotics toward synthetic bacterial communities, bacteriophage therapy, metabolite supplementation, and precision dietary strategies tailored to individual microbial and immunological profiles. The acetate supplementation study in this issue exemplifies how microbial metabolites can be leveraged therapeutically, opening the door to metabolite-based immunomodulation. Ultimately, the field must adopt a more integrative and personalized approach. Inter-individual variability in microbiota composition means that universal therapies are unlikely to succeed.

In summary, this Research Topic offers a forward-looking view of how the gut microbiota shapes immune-related inflammatory diseases. By proposing new hypotheses, embracing multi-omics integration, and advancing toward personalized microbiota-based therapies, the field is poised to make transformative contributions to human health.

